# AIDS—Bristol Virologists Reply to Dr Seale

**Published:** 1988-02

**Authors:** 


### Correspondence

**A.I.D.S.—Bristol Virologists reply to Dr Seale**

Sir,

The article by Dr John Seale in the August issue (page 66) of the Journal contains many inaccurate assertions and distortions of fact, and we wish to comment on just some of them.

We do not agree with Dr Seale's nihilistic view of viral vaccines. Successful vaccines are available for most of the common childhood exanthemata, although regretfully they are not as widely used in this country as they should be. A good vaccine is available for a lentivirus, feline leukaemia, and Dr Seale should not understate the potential of the enormous research effort addressing every aspect of the molecular biology of HIV.

Dr Seale states that "Scientists and doctors state as a fact that the AIDS virus (HIV) is fundamentally transmitted during sexual intercourse, but is also unfortunately sometimes transmitted by blood. This is highly misleading." All experts on HIV accept that virus is present in blood for the duration of infection, which is life-long, and is transmitted not only by blood transfusions and contaminated blood products such as untreated Factor VIII, but also in the smaller amounts exchanged when drug addicts share needles. The threshold dose required for HIV infection is higher than is the case with hepatitis B, so that simple needlesticks from infected patients rarely give rise to sero-conversion in healthcare workers or family contacts. HIV carriers may not necessarily have virus present in the semen at any given time, but there is direct evidence that some do and are infectious, since several women not in risk groups have become HIV antibody positive after receiving semen from a donor who was subsequently shown to be HIV antibody positive (Stewart, Tyler and Cunningham, 1985). This rules out in this instance any means of transmission associated with intercourse other than semen. The reasons for the major part played by homosexual men in the transmission of HIV infection in the United States and in other developed countries are two-fold. Firstly, before the AIDS epidemic was recognised, many of these men had considerably more sex partners than most heterosexual men and women, excluding prostitutes. Secondly and more important, assuming that semen is infectious, virus transmission is more likely to occur during homosexual activity where the fragile rectal wall of the passive partner is much more easily traumatised than is the case in heterosexual activity in the better adapted vagina. The genitalia of the active partner are not usually traumatised, so it is not easy to postulate direct blood to blood transfer during male homosexual intercourse, but this may be a less common route. Case-controlled epidemiological studies have shown that the recipient of anal intercourse is at greater risk of being infected than the active partner.

In Africa infection passes easily between both sexes. It has been postulated that this may be due to the high incidence of venereal infections in many parts of Africa. Apposition of eroded or raw inflamed areas on the genitalia will give the virus an increased chance of transmission. However, it must be emphasized that in spite of a low incidence in the heterosexual population in the West, transmission during heterosexual intercourse is a reality and has been well documented. The extent of a heterosexual epidemic in North America and Europe may well depend on a number of co-factors, the most important of which is the incidence of other sexually transmitted diseases and the sexual activities of the heterosexual population (Piot et al, 1987).

Dr Seale mentions the infectiousness of saliva. We know of only one case where infection may have been transmitted by kissing, and of no case of transmission by oro-genital or by oro-anal contact where all other factors have been excluded. Furthermore, infection has not been documented in Western families outside sexual contact or needlestick injuries and similar traumatic blood contamination. As far as transmission by insects is concerned, there is no evidence that this occurs in Africa or in a large scale study in Belle Glade, Florida, amongst a heavily HIV infected population (Communicable Diseases Centre, 1986). Transmission of HIV by insects would affect all the population but infection in Africans occurs predominantly in sexually active people. When transmission at birth and by contaminated blood transfusions and medical injections are excluded (Friedland & Klein, 1987) children and older age groups have negligible infection with HIV. We do not accept that insects have a predilection for sexually active groups while not biting the young or elderly.

To contradict Dr Seale further, there is absolutely no evidence of respiratory spread of HIV, even in African hospitals where AIDS patients are nursed under conditions of overcrowding, or in the families studied in the West. Dr Seale quotes respiratory spread by animal retroviruses but such analogies do not hold for other groups of viruses. Rubella virus is transmitted by the respiratory route whilst almost every other togavirus is spread by biting insects. Rhinoviruses cannot invade beyond the stomach whilst other members of the picornavirus family are found in the Peyer's patches of the gut.

While not wanting to suggest that any journal should suppress opinion, we are saddened to see misrepresentation of facts and wild hypothesising of no scientific credibility published in their support by an old and otherwise highly respected journal. Perhaps its Editor should consider using expert referees when there is any uncertainty about the scientific worth of proferred material! He should also ensure that it has adequate bibliography, and that previous unstated controversial assertions are backed by scientifically acceptable evidence. We do not underestimate the gravity of the AIDS epidemic, but misinformation will not help in its control and will only serve to promote irrational prejudice and hysteria.

Dr E Brown, Frenchay Hospital
Dr A P C H Roome, Public Health Laboratory, Bristol
Dr E O Caul, Public Health Laboratory, Bristol
Dr A E Jephcott, Public Health Laboratory, Bristol
Prof D C E Speller, Bristol Royal Infirmary
Dr D S Reeves, Southmead Hospital

**REFERENCES**

Communicable Diseases Centre (1986). Acquired immunodeficiency syndrome (AIDS) in Western Palm Beach County, Florida. *MMWR. Morbidity and Mortality Weekly Report.* **35** 609–612.

FREIDLAND, G. H. and KLEIN, R. S. (1987). Transmission of the human immunodeficiency virus. *New England Journal of Medicine.* **317** 1125–1135.

PIOT, P., KREISS, J. K., NDINYA-ACHOLA, J. O. *et al* (1987), Heterosexual transmission of HIV. *AIDS 1* 199–206.
STEWART, G. J., TYLER, J. P. P., CUNNINGHAM, A. L. *et al.* (1985), Transmission of Human T cell Lymphotropic virus type III (HTLV-III) by artificial insemination by donor. *Lancet* **ii** 581–585.

